# Hydrogen sulfide attenuates lung injury instigated by Bisphenol-A via suppressing inflammation and oxidative stress

**DOI:** 10.1186/s40360-022-00636-9

**Published:** 2022-12-30

**Authors:** Omayma A. R. Abo-Zaid, Fatma S. M. Moawed, Hend A. Hassan, Enas M. Moustafa

**Affiliations:** 1 Biochemistry and Molecular Biology Department, Faculty of Vet. Med, Benha University, Benha, Egypt; 2 Health Radiation Research, National Center for Radiation Research and Technology, Egyptian Atomic Energy Authority, Cairo, Egypt; 3 Radiation Biology, National Center for Radiation Research and Technology, Egyptian Atomic Energy Authority, Cairo, Egypt

**Keywords:** Bisphenol A, H_2_S, TGF-β ERK/JNK/ p38 MAPK, Lung injury

## Abstract

The xenoestrogen bisphenol A (BPA), a commonly used industrial chemical, has been linked to endocrine disruption. The point of the study was to consider the effects of chronic BPA exposure on the respiratory system of adult female rats, and the potential mitigating benefits of Sodium hydrosulfide (NaHS), a donor of hydrogen sulfide (H_2_S) administration. Detect biomarkers in Bronchoalveolar lavage fluid (BALF), including total protein content, Total cell counts, Neutrophils %, ICAM (intercellular adhesion molecule)-1 and TGF-β (Transforming growth factor beta). NaHS significantly reduced pro-inflammatory cytokines (IFN-β and MCAF,) also reduce (i.e. VCAM-1, VEGF, VIM, MMP-2, MMP-9), and reduced malondialdehyde and augmented activities of SOD and GSH-PX. Notably, H_2_S induced a marked decrease in the expression levels of p-extracellular signal-regulated protein kinase (p-ERK), p–c-Jun N-terminal kinase (p-JNK), and p-p38, H_2_S inhibits BPA-induced inflammation and injury in alveolar epithelial cells. These results suggest NaHS may prevent inflammation via the suppression of the ERK/JNK/ p-p38MAPK signaling pathway, Subsequent inhibition of inflammation, epithelial cell injury, and apoptosis may be providing insight into potential avenues for the treatment of lung injury.

**Supplementary Information:**

The online version contains supplementary material available at 10.1186/s40360-022-00636-9.

## Introduction

One of the common environmental pollutants utilized in the production of flame retardants, polyester resins, polycarbonate plastics, and epoxy resins is bisphenol A (BPA). Food and beverage packaging is made of polycarbonate plastic, and steel products like water supply pipes, cans, and bottle tops are lacquered with resins. BPA is also present in several polymers used in tooth coatings and dental sealants [1]. As polymers containing BPA may hydrolyze under high temperatures and acidic or basic conditions, leaking into food and drink containers is assumed to be the primary route of exposure in humans [2]. BPA is an estrogenic endocrine disruptor [3]. Even at extremely low concentrations, BPA's estrogen-mimicking causes a variety of health problems, including prostate [4] and breast cancer [5], as well as disruptions in reproductive function [6]. BPA also has effects that are away from the estrogenic activity, such as a disruption in the balance of inflammatory cytokines and an increase in oxidative stress. Several cytokine signals have been implicated in BPA's potential to influence and alter the immune system. As a result, disruption of cytokine signaling can cause a variety of diseases, including cancer, autoimmune disorders, inflammation, allergies, and respiratory conditions [7].

The development of common chronic illnesses including respiratory failure and lung problems is implied to be hampered. Typically, airway inflammation and reactive oxygen species (ROS) cooperate to cause lung injury [8]. Evidence suggests that individuals with lung damage suffering serious complications had considerably lower blood H_2_S levels. According to Wang et al. [9] hydrogen sulfide (H_2_S) effectively enhances respiration while lowering histological abnormalities including lung edema and leakage. One of the well-known gaseous transmitters, H_2_S, is engaged in a wide range of cellular activities, as well as physiological and pathological processes in several disorders, including respiratory ailments. H_2_S's therapeutic potential for respiratory disorders has recently attracted a lot of attention. In obstructive respiratory illness, pulmonary fibrosis, emphysema, pancreatic inflammatory/respiratory lung damage [10], liver inflammation [11], diabetic cardiomyopathy [12], and anti-cancer [13], H_2_S is an essential treatment component. We anticipated that H_2_S may modulate ROS formation, as well as metalloproteinases and adhesion molecule expression responses, MAP kinase signaling, and the release and attraction of inflammatory mediators in BPA-induced lung injury. Thus, we highlighted the function of anti-inflammatory, anti-oxidative, and feedback mechanisms in H_2_S-mediated lung protection during inflammation in the current investigation.

## Materials and methods

### Materials

Bisphenol-A (BPA, Cat # 80–05–7), NaHS (Cat # 161,527) and all other substances were bought from Sigma-Aldrich Chemical Co. in St. Louis, Missouri, USA. Lipid peroxidation is represented by MDA (malondialdehyde) is measured according to the method of Yoshioka et al. [14] through a forming thiobarbituric acid reactive substance (TBARS) reading at 532 nm. The SOD (Superoxide dismutase) and GSH-PX (glutathione peroxidase) levels were determined by Kakkar et al. [15] and Mohandas et al. [16] respectively. Rat ELISA kits were used, according to the manufacturer’s instructions, the lung VEGF (Vascular endothelial growth factor, CAT no.RRV00) R&D Systems Minneapolis, MN, lung MCAF ( monocyte chemotactic and activating factor, CSB-E07429r), The activity of ICAM (intercellular adhesion molecule -1, ab100763) Abcam, Cambridge, MA, USA, lung VCAM-1(vascular cell adhesion protein 1, CSB-E07275r), the level of, IFN-β (Interferon-β, CSB-E04845r), VIM (vimentin, CSB-E14029r). Rat TGF-β1 (Transforming Growth Factor β1, CSB-E04727r), MMP-2 and MMP-9 (matrix metalloproteinase 2 and 9, CSB-E07411r, CSB-E08008r) and TIMP-1 (Tissue inhibitors of metalloproteinase 1, CSB-E08005r) levels were measured using ELISA kits supplied by CUSABIO® Technology, LLC. The samples were determined using an ELISA plate reader from BioTek, Vermont, U.S.A.

### Experimental animals

In this study, 5 weeks-old Wister female rats (150 ± 20 g) were used, obtained from the National Centre for Radiation Research and Technology (NCCRT), Cairo, Egypt. Rats were kept in a pathogen-free environment with specialized air conditioning, a 12:12 daylight/darkness cycle, and unrestricted access to food and drink. The experimental protocol was carried out according to *ARRIVE* guidelines (serial: 18–2019) as approved by the Animal Ethical Committees of Benha University with an ethical approval number (BUFVTM05-01–22).

### Experimental protocol

After a one-week acclimatization period, the rats were randomly assigned to four groups (8 rats/group) and caged separately as follows: Group I (Control) served as the control untreated group, and animals in group II Bisphenol -A (BPA) were injected IP with BPA (150 mg/kg/ day) dissolved in corn oil for 4 weeks [17], group III (H_2_S) rats were treated with H_2_S donor NaHS intraperitoneally (IP) at a daily dose of 5 mg/kg/day dissolved in deionized water for 6 weeks [18], Group IV (BPA + H2S) rats were injected with BPA as group II and H_2_S donor NaHS as group III for 6 weeks.

At the end of the experiment, animals were sacrificed by cervical dislocation. The chest was opened and fresh lung specimens were taken. Dissected lung tissue samples were collected and stored at ‒80 °C for further molecular and biochemical analysis. The Bronchoalveolar lavage fluid (BALF) was performed with 500 µl of saline via an insertion tube into the trachea. The BAL fluid was extracted to detect biomarkers, including total protein content, which was assayed to evaluate the integrity of the alveolar-capillary membrane barrier and to evaluate lung vascular outflow, determined using the Bio-Rad protein assay kit. Total cell counts were determined using a hemocytometer. Differential cell counts were determined on smear slides stained with He-color (EMD Chemicals). Neutrophils % was calculated as their ratio × 100 divided by the total number of cells in each BALF sample.

### Western blot analysis

Pieces of lung tissue were homogenized in a cold homogenizer tube containing 2 ml of homogenization buffer for the measurement of ERK1/2, p-JNK, and p-p38. Thermo-scientific provided the ERK1/2, p-JNK, and p-p38 primary antibodies. The published data were produced using β-actin protein expression normalization (as a housekeeping protein), as described in Bradford [19]. The bands was cut from the membrane and didn’t keep an image before cropping. The image with a full length marker are shown from highest to the lowest molecular weight bands in the supplementary file.

### Statistical analysis

All parameters are expressed as the mean ± SD. Using one-way ANOVA and the Bonferroni multiple comparison tests, the findings were statistically examined. The criterion for significance was a *p* < 0.05. Analysis was performed using SPSS 20 software package (Analytical Software, USA).

## Results

### Lipid peroxidation MDA, Superoxide Dismutase (SOD) and Glutathione Peroxidase (GSPX) levels in lung rat tissues

**Fig. 1:**
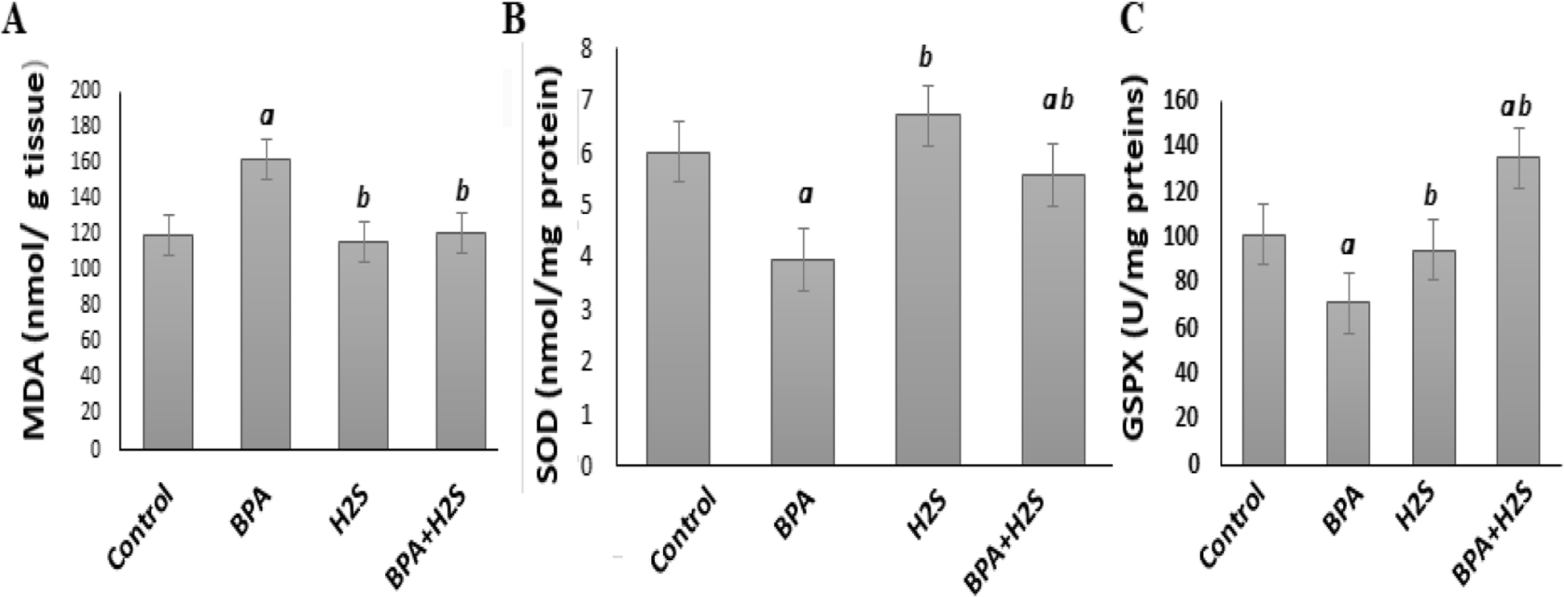
MDA, SOD and GSPX levels in lung tissues of rats exposed to BPA/H_2_S.The results are presented as the mean ± SE (*n* = 8 rats). **a)** Significant difference versus control group at *P* ≤ 0.05. **b)** Significant difference versus BPA group at *P* ≤ 0.05

Figure 1 demonstrates that in the rats given BPA injections, there was a discernible rise in lipid peroxidation (MDA) levels and a decline in SOD and GSPX levels. On the other hand, H_2_S injection significantly modulated the levels of MDA and SOD and GSPX activities.

### Vascular endothelial growth factor (VEGF), monocyte chemotactic and activating factor (MCAF) and Cellular adhesion molecules VCAM levels in BAL fluid lung rats

**Fig. 2:**
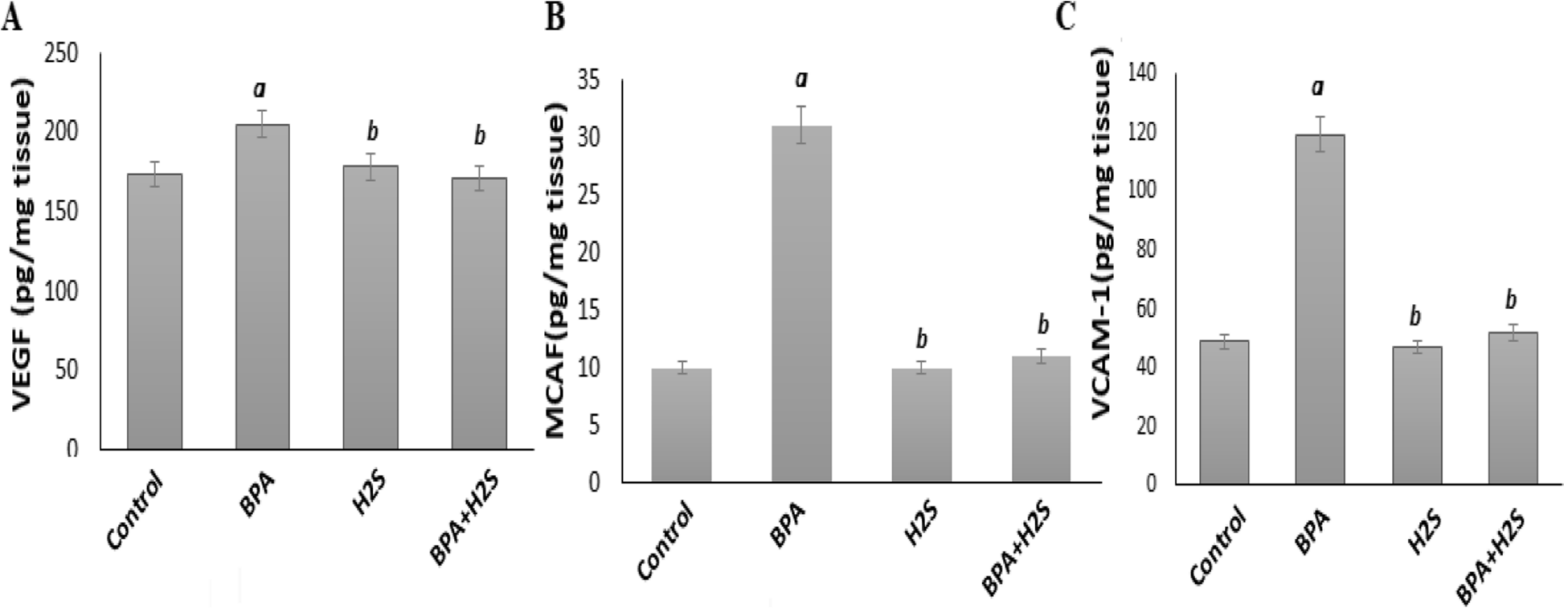
VEGF, MCAF and VCAM-1 (ng/ml) in lung tissue of rats exposed to BPA/H_2_S The results are presented as the mean ± SE (*n* = 8 rats). **a** Significant difference versus control group at *P* ≤ 0.05. **b)** Significant difference versus BPA group at *P* ≤ 0.05

According to the findings in Fig. 2, rats exposed to BPA experienced a considerable rise in their levels of VEGF, MCAF, and VCAM-1 in comparison to the control group. VEGF, MCAF, and VCAM-1 level are significantly lower in the H_2_S-treated groups compared to the BPA group.

### Levels of interferon β (IFN β) and Vimintine (VIM) in lung rats tissue

**Fig. 3:**
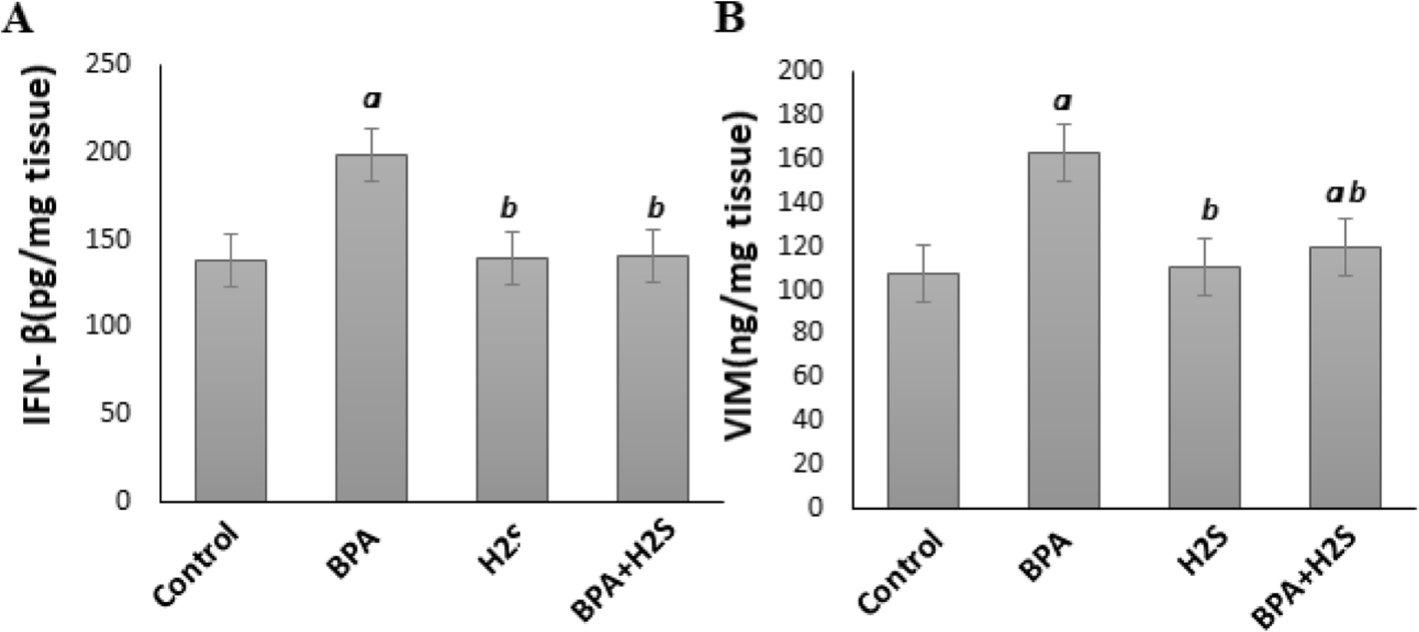
IFN-B and VIM levels in lung tissues of rats exposed to BPA/H_2_S.The results are presented as the mean ± SE (*n* = 8 rats). **a)** Significant difference versus control group at *P* ≤ 0.05. **b** Significant difference versus BPA group at *P* ≤ 0.05

According to the findings in Fig. 3, rats exposed to BPA showed a significant rise in both IFN-β and VIM levels compared to the corresponding levels in the control group. However, H_2_S injection significantly lowered the levels of INFβ and VIM compared to the BPA group.

### Levels of Metalloproteinase (MMP-2 and MMP-9) and tissue inhibitor Metalloproteinase—1 (TIMP-1) in lung rat tissue

**Fig. 4:**
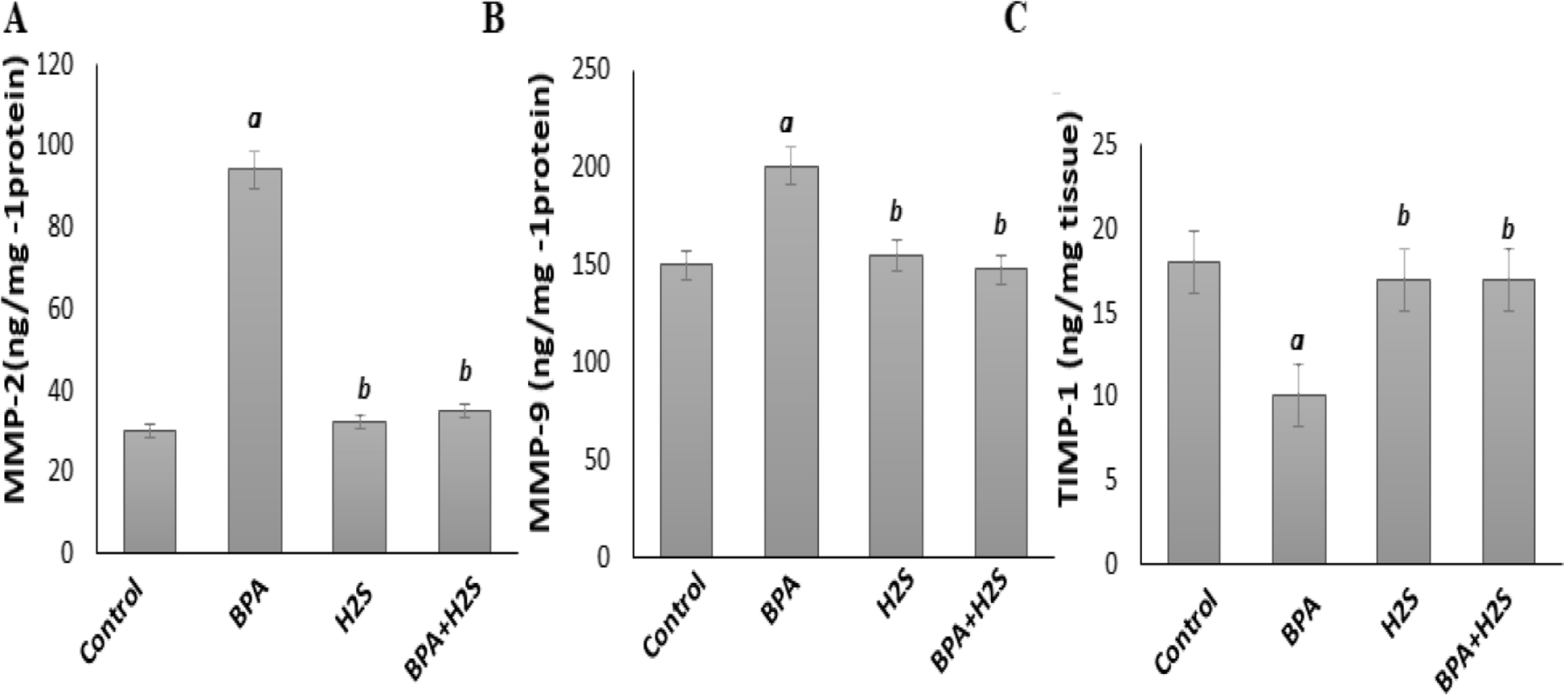
MMP-2, MMP-9 and TIMP-1 levels in lung tissues of rats exposed to BPA/H_2_S. The results are presented as the mean ± SE (*n* = 8 rats). a) Significant difference versus control group at *P* ≤ 0.05. b) Significant difference versus BPA group at *P* ≤ 0.05

As shown in Fig. 4 BPA significantly increased the levels of MMP-2, MMP-9, and TIMP-1; *p* < 0.05) compared to the control group.Meanwhile, there is a substantial reduction in MMP-2, MMP-9, and TIMP-1 in H_2_S-treated group compared with BPA group,

### Levels of Inflammatory markers (Total cells and TGF-B1) in lung BALF

**Fig. 5:**
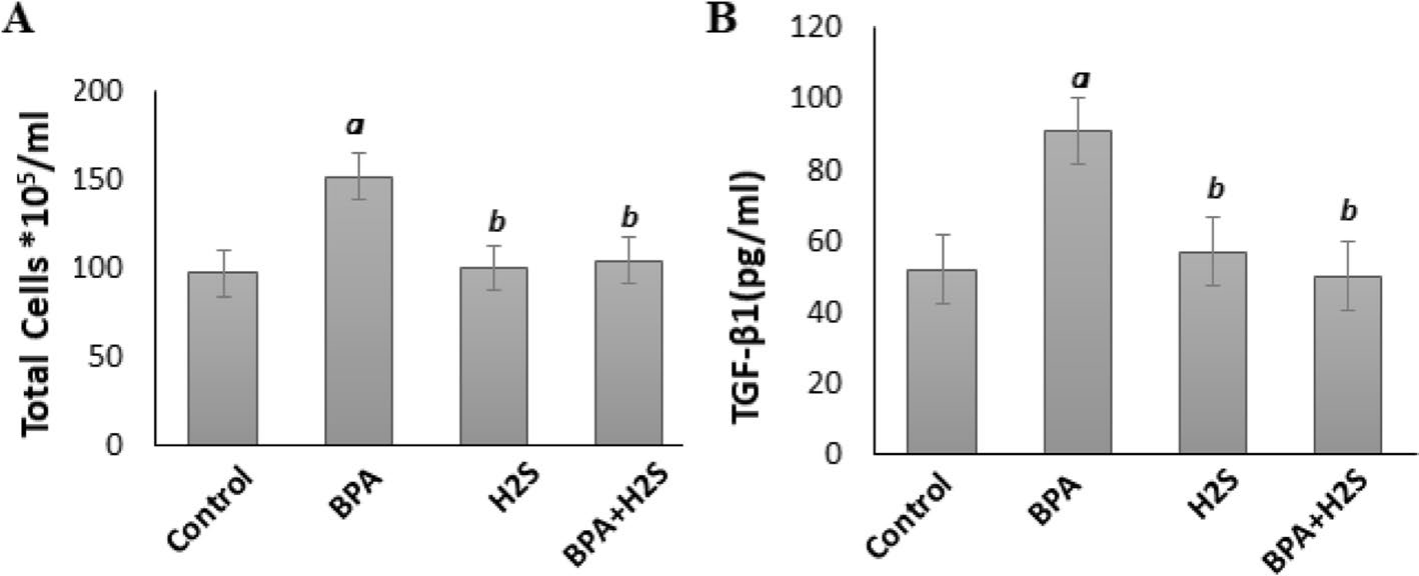
Total cells*105/ml and TGF-B1 (pg/ml) levels in lung BALF of rats exposed to IR/BPA/H_2_S. The results are presented as the mean ± SE (*n* = 8 rats). a) Significant difference versus control group at *P* ≤ 0.05. b) Significant difference versus BPA group at *P* ≤ 0.05

The findings in Fig. 5 demonstrated that rats exposed to BPA showed a substantial rise in both total cells and TGF-β1 levels compared to the corresponding values in the control group. When compared to the BPA group, the H_2_S group has a significantly lower total cell count and TGF- β levels.

### Levels of P-JNK, P38MPAK and P-ERK by Western blot

**Fig. 6:**
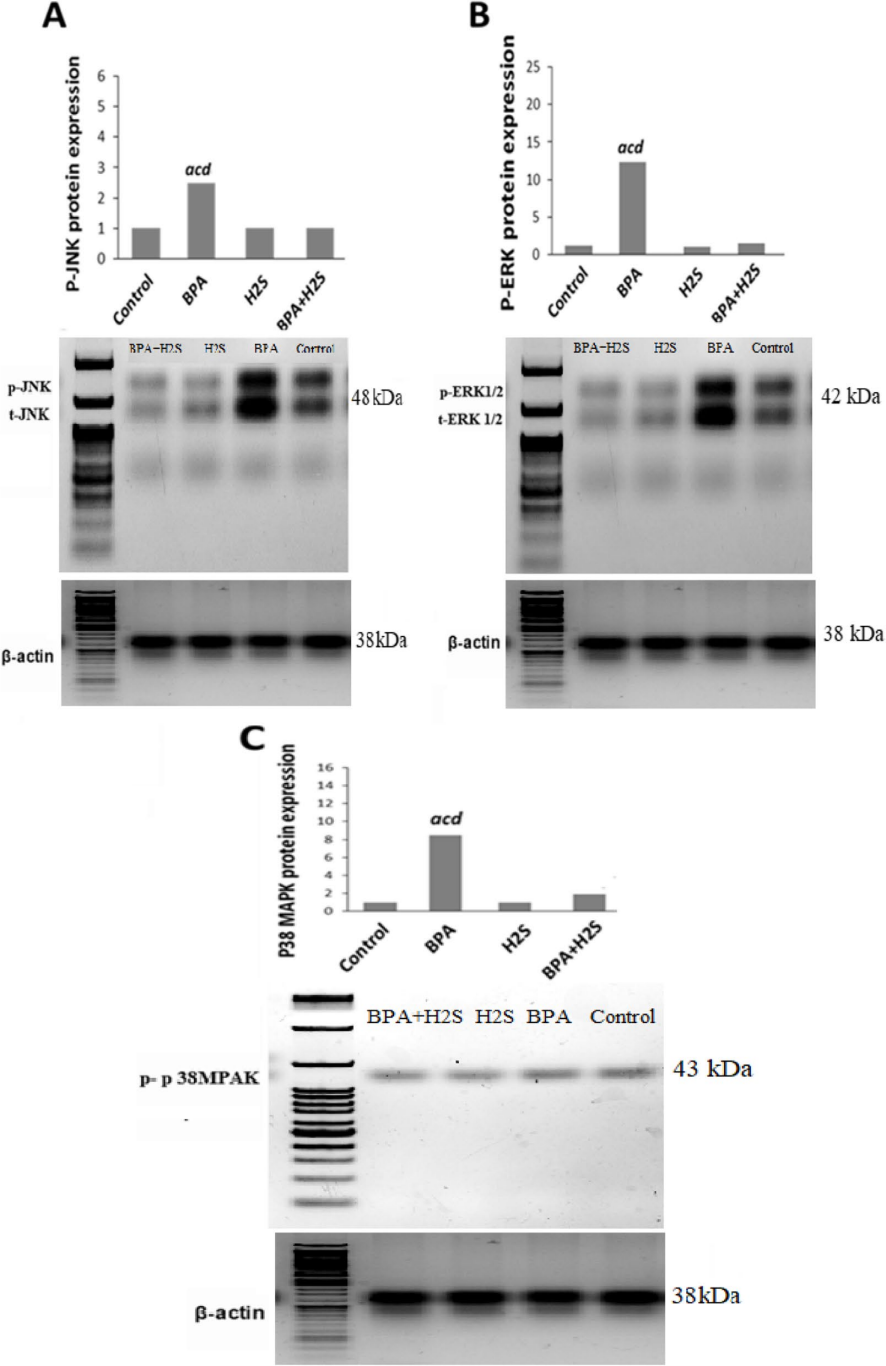
Relative protein expression levels of (**A**) t/p-JNK, (B) t/p-ERK1/2, and (**c**) p38MPAK fold changes in lung tissue cells in different studied groups as distinguished by western blot analysis. Relative quantification of western blot analysis is presented on SDS-PAGE 1) BPA + H2S, 2) H_2_S, 3) BPA 4) Control groups. The results are presented as the mean ± SE (*n* = 8 rats). a) Significant difference versus control group at *P* ≤ 0.05. b) Significant difference versus BPA group at *P* ≤ 0.05. The bands was cut from the membrane and didn’t keep an image before cropping. The image with a full length marker are shown from highest to the lowest molecular weight bands in the supplementary file

According to the results of this study, lung tissue cells treated with BPA had increased relative protein expression levels of P-JNK, P38MPAK, and P-ERK, which could be identified by western blot analysis. According to Fig. 6, it was observed that groups treated with H_2_S exhibited a lower level of P-JNK, P38MPAK, and P-ERK than groups exposed to BPA.

### Levels of Protein, %Netrophils and ICAM -1 in lung BALF of rats

**Fig. 7:**
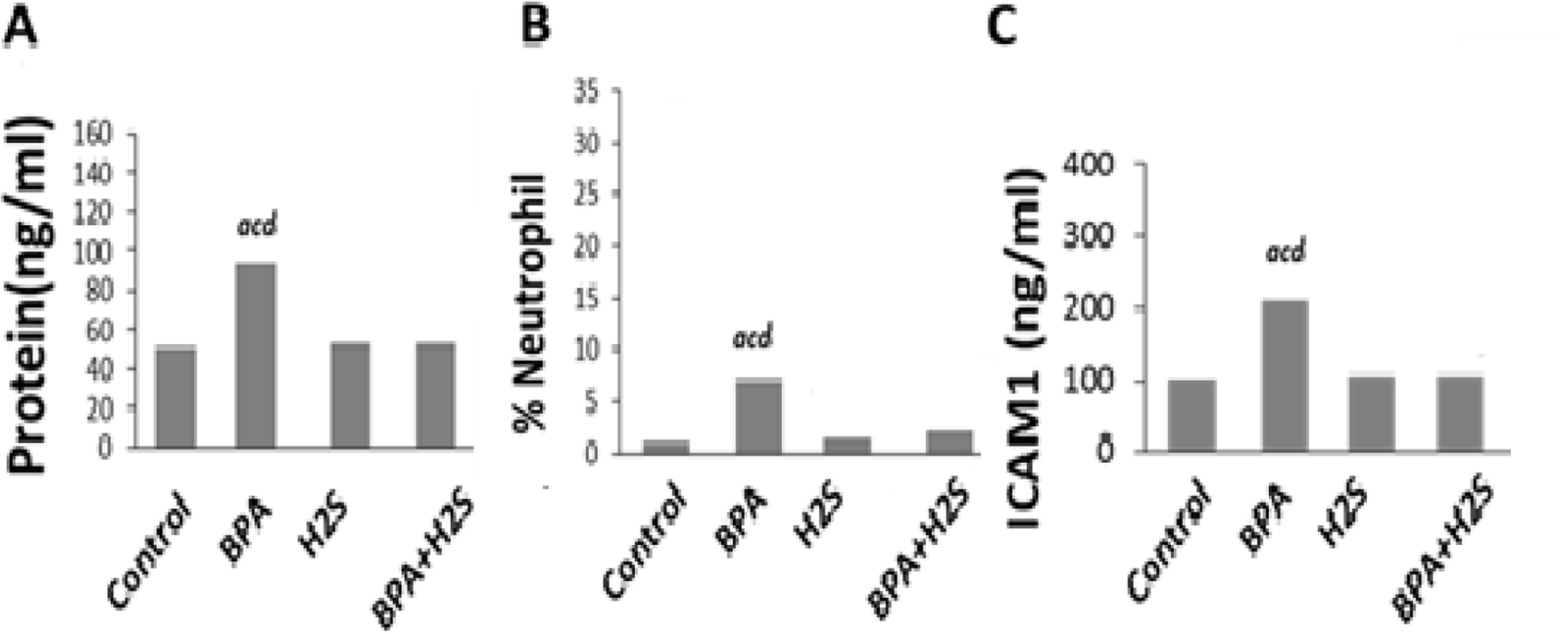
Protein concentration, %Netrophils and ICAM-1 level in BALF fluid of rats exposed to BPA/H_2_S. The results are presented as the mean ± SE (*n* = 8 rats). **a** Significant difference versus control group at *P* ≤ 0.05. **b** Significant difference versus BPA group at *P* ≤ 0.05

The results presented in Fig. 7 showed that the exposure of rats to BPA induced a significant increase in the level of Protein, % Neutrophils and ICAM-1 compared with their respective values in the control group. There is a significant decrease in Protein, %Netrophils and ICAM-1 in groups treated with H_2_S compared to the BPA group.

## Discussion

Lung infection-related lung damage is still a serious issue and is linked to high morbidity and death rates. There are currently no particular treatments available to improve lung damage, despite current medical advancements and supportive care. In this regard, it has been shown that breathed H_2_S in a modest dosage can effectively prevent lung harm in a mouse model of pulmonary inflammation [20]. The underlying molecular mechanisms in this model influenced by H_2_S are still unknown. In the current work, we demonstrate for the first time that H_2_S mediates this protection by inhibiting BPA-induced inflammatory and oxidative processes, which may then lead to the observed organ protection.

In a mouse model of acute lung damage, it has been demonstrated that exposure to H_2_S prevents the development of acute lung injury [20]. In the current study, BPA administration induced a deleterious effect by increasing the pro-inflammatory markers (TGF-1β, VEGF, MCAF, VCAM-1, IFN β and VIM) as well as exaggerating oxidative stress through inhibition of the antioxidant enzyme activities of SOD and GSPx. In addition, the activity of MMP9 and MMP2 was enhanced and TIMP-2 was decreased. These results are in agreement with the findings of Kim et al. [21] where Bleomycin Induced-Pulmonary Fibrosis in rats is associated with increased transcription of MMP-9 and MMP-2.

Inflammatory response, oxidative stress, and metalloproteinases have all been associated with lung damage induced by BPA treatment [21, 22]. It has been demonstrated that lung damage is mediated by neutrophil migration into lung tissue and the production of pro-inflammatory cytokines. In our model, exposure to BPA caused significant transmigration of neutrophils into the bronchoalveolar fluid. This was accompanied by an increase in the secretion of TGF-1, IFN, ICAM-1, MCP-1, and VCAM-1, as well as an exaggeration of oxidative stress due to the inhibition of SOD and GSPx, two antioxidant enzymes. TIMP-2 activity was also lowered, whereas MMP9 and MMP2 activities were increased.

On the other hand, in the current study, the inflammatory biomarkers (TGF-1 and IFN as well as ICAM-1, MCP-1, and VCAM-1) were modified by H_2_S exposure. These results are consistent with studies demonstrating the anti-inflammatory properties of H_2_S in *vivo* lung injury models like pulmonary inflammation [23, 24], mechanical ventilation [25], hyperoxia [26], or acute pancreatitis [27], reflecting that H_2_S-induced lung protection is mediated by anti-inflammatory and antioxidant effects.

Increased ROS generation is a hallmark of acute lung injury produced by BPA exposure, and it has been demonstrated in several models that its inhibition can protect lungs from BPA-induced lung damage [22]. H_2_S could prevent ROS production by scavenging free radicals, regulating oxidative signaling pathways and reducing inflammation. In a mouse model of hyperoxia, ROS were decreased by the administration of H_2_S, which was associated with the suppression of lung injury. Similarly, it was found that H_2_S administration in activated epithelial A549 cells, endothelial HUVEC cells, RAW 264.7 macrophages, PC12, and H9c2 cells, decreased ROS production and consequently suppressed the inflammatory response [24].

Disruption of the blood-air barrier, which is mainly composed of the alveolar epithelium, capillary endothelium, extracellular matrix (ECM), alveolar macrophages, and other cells, is regarded as a central event in the pathogenesis of lung injury. The matrix metalloproteinases (MMPs) are thought to be the primary physiological mediators of ECM degradation. Under normal conditions, MMPs are secreted from cell's inactive forms (pro-MMPs); however, the majority of MMPs can be activated and significantly secreted during systemic inflammatory responses and tissue damage, such as lung injury, which was characterized by the disruption of the blood-air barrier [28]. In the current work, we showed that BPA greatly elevated MMP-2 and MMP-9 activities, which prompted the critical contributions of MMP-2 and MMP-9 to the development of BPA-induced lung damage. TIMP1, the inhibitor of MMPs, was also elevated, which indicated that the body may have also undergone some limited tissue repair. The investigation showed that the elevated expression of MMP-2 and MMP-9 was also seen in the exposure to other hazardous compounds supporting our findings [29].

The MAPK signaling pathway is another potential player in the H_2_S-induced lung protection that has been reported. It has been suggested that controlling MAPK activity might increase lung damage by mediating inflammatory and/or oxidative responses to viral insults. H_2_S injection has also been characterized as inhibiting MAPK signaling. In a PC-12 cell model of hypoxia-induced injury, H_2_S treatment decreased the expression of p38 and ERK 1/2, and in a rat model of ischemia–reperfusion, H_2_S protected endothelial cell damage by inhibiting p38 and JNK signaling. Our findings indicated that the modulation of pJNK, pERK1/2 and p38 MAPK expression was significantly affected by BPA. However, the injection of H_2_S dramatically decreased the phosphorylation of the pJNK, pERK1/2 and p38 MAPK expression. In *vitro* models of LPS-induced inflammation or cobalt chloride-induced hypoxia, it was found that therapy with H_2_S can block p38 MAPK activation and provide cellular protection [30]. Furthermore, Sivarajah et al. demonstrated that the observed cardioprotective effects of H_2_S in a rat model of myocardial ischemia–reperfusion injury were induced by a decrease in p38 phosphorylation [31], indicating that H_2_S-related decreases in pp38 may be involved in our model's protection from BPA-induced lung injury.

According to the findings of the study, lung damage caused by BPA can be attenuated by administering H_2_S, which affects the levels of proinflammatory cytokines (TFGF-1 and IFN-β), chemokines (VEGF, MCAF, VCAM-1), metalloproteinases (MMP-2 and MMP9), P38-MAPK protein expression, and oxidative stress biomarkers. Additionally, the anti-inflammatory effects of H_2_S-mediated lung protection are caused by preventing oxidative stress biomarkers mediated by inhibition of MMP-2 & MMP-9 and p38 MAPK signaling pathways, even though the results of the current study do not reveal the rate of contribution or a potential interaction between the signaling pathways.

## Supplementary information

**Additional file1.**

## Data Availability

The corresponding author (Fatma S.M. Moawed, fatmasearch5@yahoo.com) will provide the datasets produced and/or analysed during the current work upon reasonable request.
