# Supplementary material for: Hydrogen sulfide attenuates lung injury instigated by Bisphenol-A via suppressing inflammation and oxidative stress
Source: BMC Pharmacol Toxicol. 2022 Dec 30;23:98. doi: 10.1186/s40360-022-00636-9 (PMC9805095; doi:10.1186/s40360-022-00636-9)
Supplement: Supplementary file 1 [file 40360_2022_636_MOESM1_ESM.docx]

KDa M BPA+H2S H2S BPA Control

30
40
50
80
140

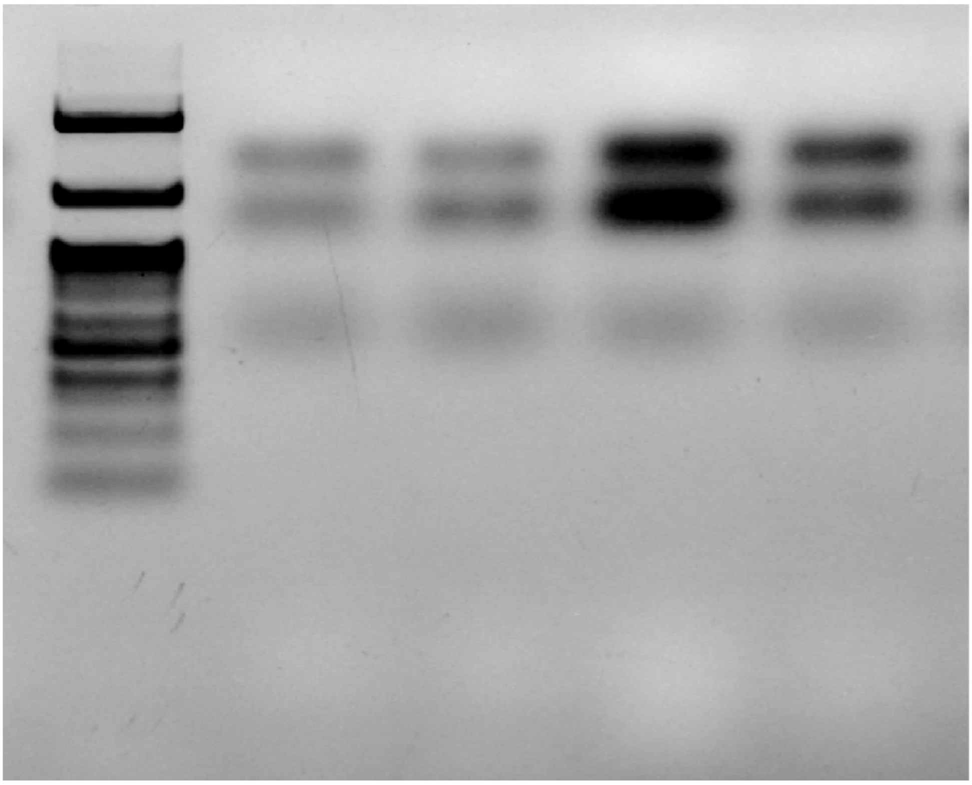

ERK1/2

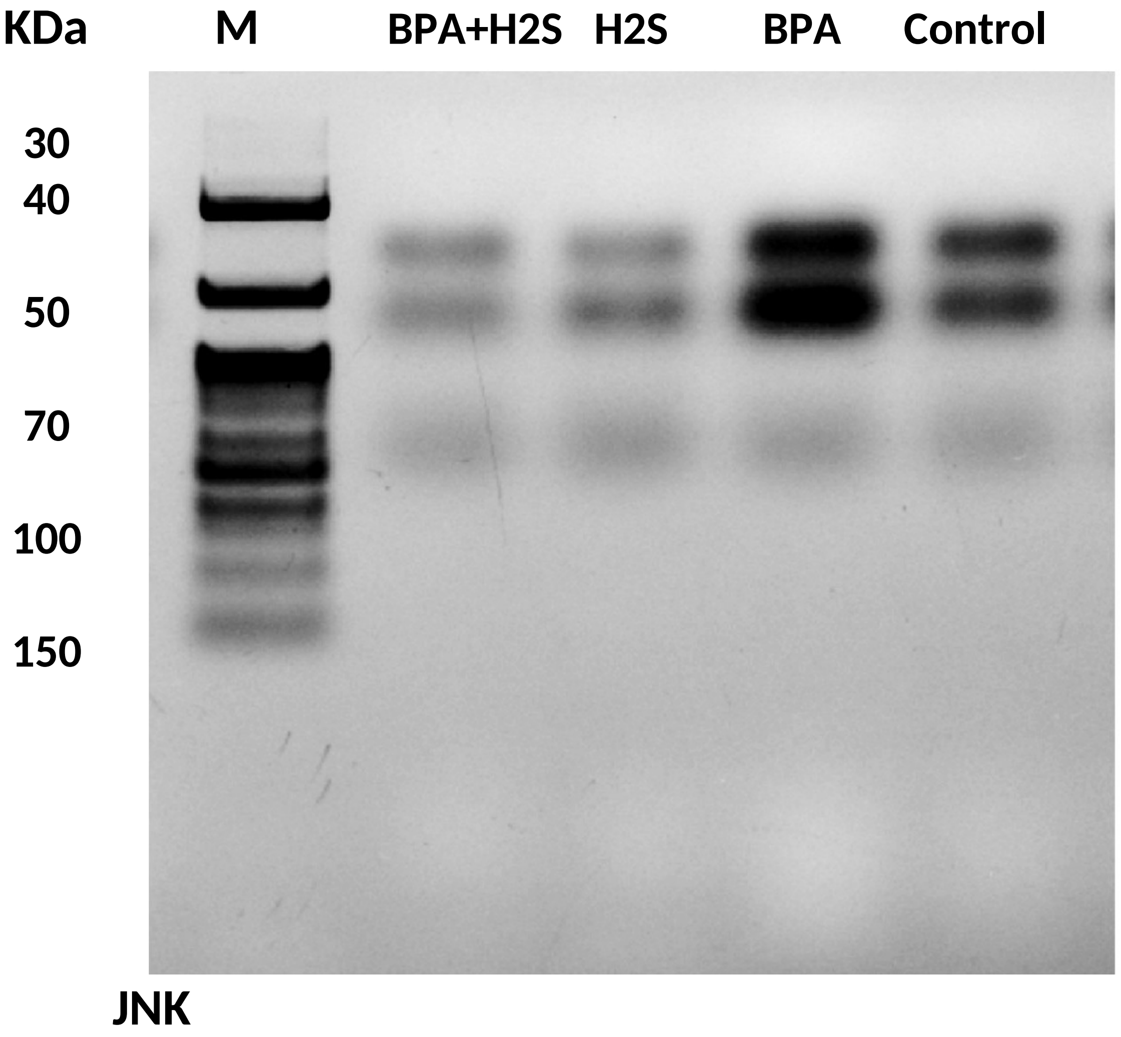
KDa
M
BPA+H2S
H2S
BPA
Control
30
40
50
70
100
150
JNK

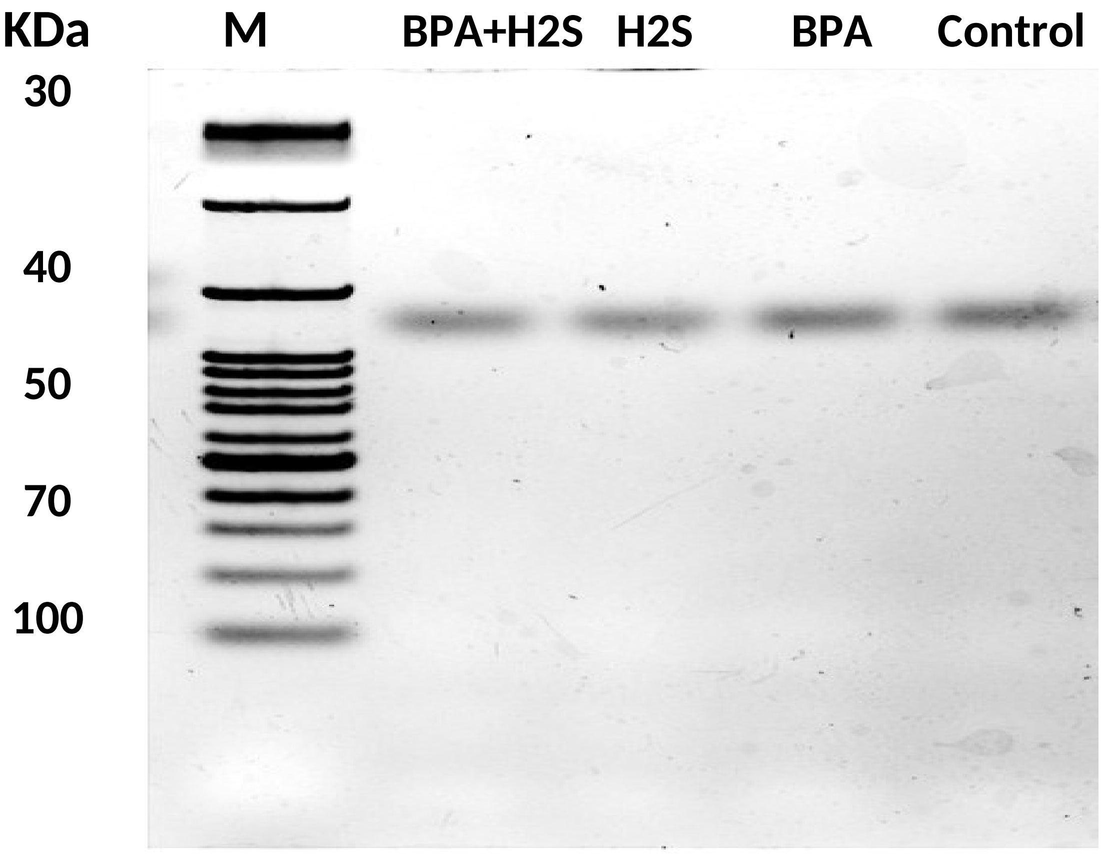

P-38MPAK

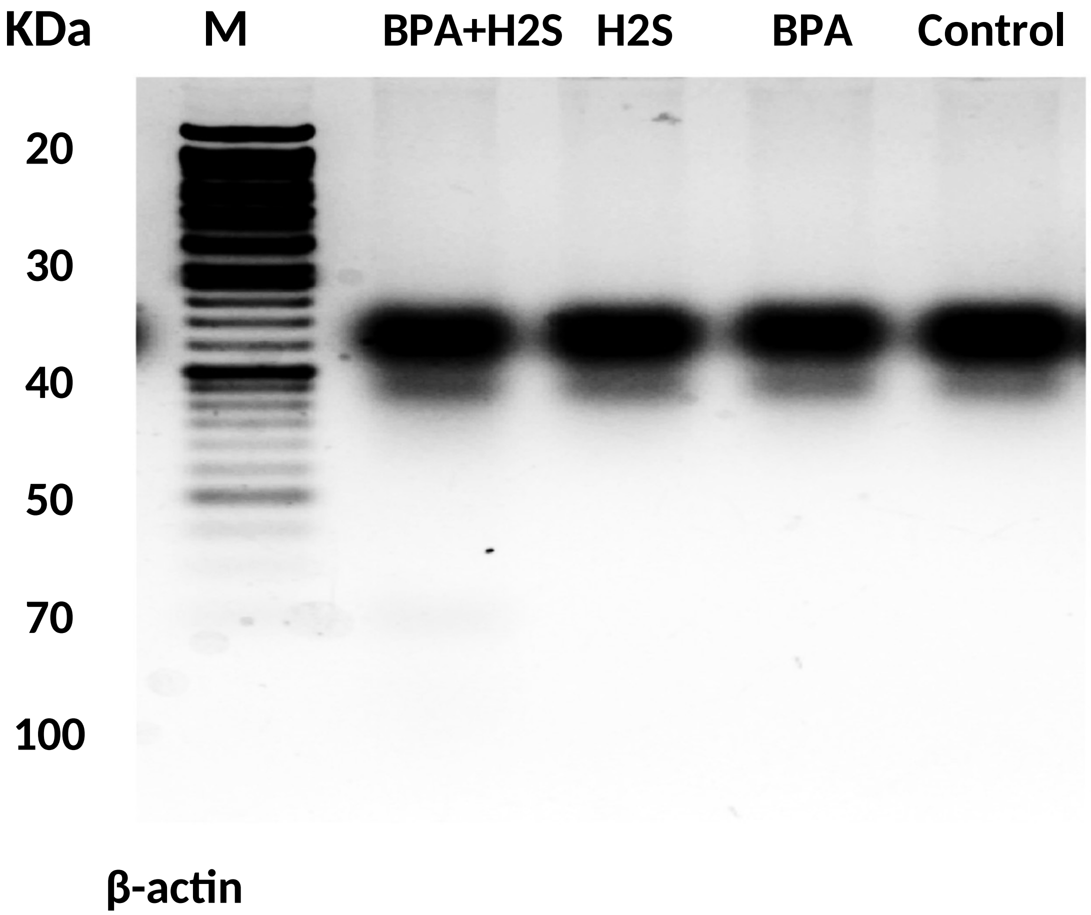
KDa
M
BPA+H2S
H2S
BPA
Control
20
30
40
50
70
100
β-actin
